# Behavioral effects of congenital ventromedial prefrontal cortex malformation

**DOI:** 10.1186/1471-2377-11-151

**Published:** 2011-12-02

**Authors:** Aaron D Boes, Amanda Hornaday Grafft, Charuta Joshi, Nathaniel A Chuang, Peg Nopoulos, Steven W Anderson

**Affiliations:** 1 Department of Pediatric Neurology, Massachusetts General Hospital, Harvard University. MC WACC 8-835, 55 Fruit Street. Boston, MA 02114. USA; 2 Departments of Neurology and Psychology, University of Iowa. 200 Hawkins Drive, 2007 RCP, Iowa City, IA 52242. USA; 3 Department of Pediatric Neurology, University of Iowa. 2506 John Colloton Pavilion, 200 Hawkins Drive, Iowa City, IA 52242. USA; 4 Department of Neuroradiology, Rady Children's Hospital of San Diego. 3020 Children's Way. MC 5101, San Diego, CA 92123. USA; 5 Department of Psychiatry, University of Iowa. 200 Hawkins Drive, W285 GH, Iowa City, Iowa 52242. USA; 6 Department of Neurology, University of Iowa. 200 Hawkins Drive, 2007 RCP, Iowa City, IA 52242. USA

## Abstract

**Background:**

A detailed behavioral profile associated with focal congenital malformation of the ventromedial prefrontal cortex (vmPFC) has not been reported previously. Here we describe a 14 year-old boy, B.W., with neurological and psychiatric sequelae stemming from focal cortical malformation of the left vmPFC.

**Case Presentation:**

B.W.'s behavior has been characterized through extensive review Patience of clinical and personal records along with behavioral and neuropsychological testing. A central feature of the behavioral profile is severe antisocial behavior. He is aggressive, manipulative, and callous; features consistent with psychopathy. Other problems include: egocentricity, impulsivity, hyperactivity, lack of empathy, lack of respect for authority, impaired moral judgment, an inability to plan ahead, and poor frustration tolerance.

**Conclusions:**

The vmPFC has a profound contribution to the development of human prosocial behavior. B.W. demonstrates how a congenital lesion to this cortical region severely disrupts this process.

## Background

The last twenty years have witnessed a surge of discoveries about the ventromedial prefrontal cortex, a brain region centered at the ventral (orbital) and medial surfaces of the prefrontal lobe. Lesion and functional imaging studies have combined to highlight the critical role of the vmPFC in highly valued human processes, including: decision making [1], emotion regulation [2], moral judgment [3], empathy [4], and impulse control [5]. Dysfunction of the vmPFC has been implicated in certain psychiatric disorders, including pathological antisocial behavior (e.g. developmental psychopathy [6], conduct disorder [7], antisocial personality disorder [8]) as well as mood disorders [9] and impulse control disorders (reviewed in [10]).

Much of the foundation for what is known about the vmPFC has come from rigorous characterization of the behavioral profile following adult-onset focal lesions of the vmPFC [11-22]. These individuals typically perform normally on a standard neurological exam (language, memory, general intelligence, sensorimotor), but display dramatic disturbances in personality and real world behavior that are more severe than what is seen after injury to other sectors of the prefrontal lobe [23]. Core deficits include: egocentricity, a lack of empathy, poor decision-making, socially inappropriate behavior, and an overall blunted emotional experience with the exception of emotional outbursts that are often frustration-induced. These individuals tend to be self-indulgent, impulsive, and lack insight with regard to how their behavior impacts those around them. This syndrome of acquired social maladjustment following vmPFC injury has many similarities to the affective and interpersonal style seen in developmental psychopathy [24,25], prompting descriptors such as pseudopsychopathy [12], or acquired sociopathy [11,22].

A key limitation to studying adults with vmPFC injury is that it only offers insight into what the vmPFC contributes to adult behavior. Of even greater relevance for understanding the role of the vmPFC in human behavior and in psychiatric disease is to understand how the vmPFC contributes to development. Studies of early-onset focal insult to the vmPFC are particularly instructive, though vanishingly rare. There are just a few case reports of adults who acquired damage to the vmPFC and/or adjacent cortices early in life [21,26-28] and one article that describes children with early-onset vmPFC injury [28]. These individuals tend to have severe antisocial behavior and impaired moral judgment, a behavioral phenotype similar to but more severe than their adult-onset counterparts [26].

In this case report we describe a 14 year-old boy with neurological and psychiatric sequelae that are presumed to stem directly from anomalous cortical development of the left vmPFC. This case is noteworthy in being the first detailed behavioral profile associated with a focal congenital malformation of the vmPFC. The advantage of studying behavior in someone with a congenital lesion is that it eliminates the confounding possibility that life experience with a functional vmPFC is influencing behavior through consolidated memories in widespread networks in unaffected brain regions. This is a concern of behavioral studies of late-onset vmPFC lesions. This case is also noteworthy for being only the second report of behavioral manifestations of vmPFC lesions in the pediatric population (also see [28]). This allows real-time behavioral testing during childhood as opposed to post-hoc recollection of childhood behavior, which is more susceptible to recall bias. By observing this patient's behavioral profile and specific deficits we have a window into what the vmPFC contributes to different stages of human development.

## Case Presentation

B.W. is a 14 year-old right-handed Caucasian boy. He lives in a small Midwest town with his biological parents and 5 full biological siblings of which he was the second born. His parents are both college-educated; his father is an engineer and his mother a homemaker and former teacher. Through several interactions with the parents they have been regarded as pleasant, caring, and intelligent. The family history is notable for the absence of anyone with seizures or behavioral problems. The only psychiatric history in the family is mild generalized anxiety symptoms in the father and B.W.'s older sister that have not required treatment. B.W.' s mother had routine prenatal care and he was delivered at term without complications. B.W. developed normally and met all major developmental milestones on time. He had an unremarkable medical history until four years of age, at which time he began to have discrete fifteen second episodes of unresponsiveness characterized by facial flushing, hand-wringing, increased heart rate, and incoherent fearful speech followed by laughing. These episodes occurred every 30 to 40 minutes for a few days, prompting evaluation by a pediatric neurologist. A diagnostic workup included routine laboratory tests, a head computed tomography (CT), a brain magnetic resonance imaging (MRI) scan, and an electroencephalogram (EEG). The EEG showed a seizure tendency and one seizure was captured with a focal origin (focal site not reported). Other study results were reportedly within normal limits. He was started on divalproate (Depakote) with complete resolution of seizures. A subsequent review of the locally obtained brain MRI seven years later would reveal a vmPFC malformation (described below).

At age six B.W.'s parents reported the onset of defiance at home and at school, including: stealing, lying, aggression, rage, rude language, and disobedience. His parents referred to this as his 'contraband' period because he would consistently bring prohibited items to school (e.g. a pocketknife). He also stole cookies and would sell them to peers. The parents were very concerned about this behavior because it did not seem characteristic of B.W.'s previous temperament. Moreover, neither parent nor any sibling of B.W. had similar behavioral problems. He was seen by a child psychologist and diagnosed with oppositional defiant disorder and started counseling, which was discontinued after a few visits.

During ages seven to nine B.W.'s parents describe a 'cause and effect problem' in which he would behave badly and be punished and the following day would engage in the same behavior that led to the punishment. Along with his lack of response toward punishment, B.W. was impulsive and showed a lack of respect toward authority, including teachers and parents. In an effort to provide greater structure and discipline than the school could provide the parents decided to begin home-schooling B.W. and his siblings when he was nine years old (fourth grade). At the onset of home-schooling the mother noted a stark contrast between B.W. relative to his well-behaved siblings. Despite behavioral problems and lack of self-motivation he was noted to be intelligent and academically capable. The following year a child psychiatrist diagnosed B.W. with attention deficit hyperactivity disorder and bipolar disorder, for which he was prescribed carbemazepine, topiramate, and dexmethylphenidate. Counseling was again attempted briefly without effect.

At age 11 B.W. presented to the emergency room of a large tertiary care center with his mother for suicidal ideation. While at a nearby shopping mall he expressed feelings of hopelessness, unworthiness, and wanting "to kill myself... I would cut or burn myself." The talk of suicide had been ongoing for two months and had been accompanied by suicidal gestures such as jumping from a second story deck onto a trampoline and a superficial laceration to the left hand because "I wanted to kill myself." Along with the suicidal gestures the parents were alarmed about escalating aggression, destructive behavior, wandering off, and hypersexual behavior that included masturbation, accessing porn sites on the web, and asking younger peers to disrobe in a domineering manner (despite being pre-pubescent at the time). During the admission interview he reported that he had been hearing voices at night from God and the devil motivating him to do good and bad things, respectively.

B.W. was hospitalized for one week on the child psychiatry service (at which point he become known to the author, A.D.B.). He underwent extensive neuropsychological and psychiatric evaluation. His verbal and nonverbal intellectual abilities were measured using the Wechsler Abbreviated Scale of Intelligence and found to be within the average and high average range, respectively (verbal IQ 94, performance IQ 116)[29]. He endorsed extremely high levels of depression and anxiety symptoms on the Beck Depression and Anxiety Inventories for Youth (T score values of 81 for anxiety and 84 for depression), but these symptoms appeared incongruent with his mood and affect [30]. While observed on the inpatient unit B.W. displayed neutral affect though rarely had outbursts that were induced by frustration. His outward manifestations of depression were transient and thought to be manipulative in nature toward the staff and parents. On projective testing using Rotter's Incomplete Sentences [31] B.W. expressed his anger at being told no when people prevented him from carrying out his desires, which often focused on acquiring objects. My biggest problem is..."I can't get a cell phone". The worst thing that ever happened to me was..."my mom and dad saying no to it", and my mother should... "let me do some stuff that I want". The pediatric neuropsychologist conducting the tests reported that B.W. attempted to manipulate and control the interview and was angered when requests for items belonging to the examiner were denied.

It became apparent that B.W. wanted to stay in the hospital for an extended period, ("at least a month") for reasons that were not clear. When he spoke with the staff and with his parents he persistently bargained for items that would improve his mood when he returned home. He was fixated on getting a cellular phone, an electric scooter, and his own bedroom. The parents and staff became suspicious that the mood symptoms and psychosis that prompted hospitalization may have been contrived for these secondary gains. This deception may have contributed to the discrepancy in B.W,'s high subjective reports of depression and anxiety that did not match his neutral affect. The parents confirmed a long history of being manipulative in multiple settings, often for the purpose of acquiring toys or avoiding punishment. For example, he would persuade his friends to allow him to spend the night at their house and would return home with their prized goods (e.g. toys, clothes, shoes). He had a history of persuading his friends to steal money from their parent's purse or wallet for him. While hospitalized B.W. lied on occasion in an attempt to receive rewards such as small toys and credit for playing video games that he did not earn. He also attempted to spread lies among the staff that his parents were not comfortable taking him home as his discharge approached. He was given the following psychiatric diagnoses according the diagnostic and statistical manual of mental disorders (DSM-IV): oppositional defiant disorder, attention deficit and hyperactivity disorder (ADHD) combined subtype, and mood disorder not otherwise specified [32]. None of these diagnoses captured what was believed to be the core deficit, his ability and willingness to manipulate others as he pursued his own interests, which, at this point in his life, focused on the acquisition of prized items such as a cellular phone. Months later during a follow-up psychiatry visit he reported that he never intended to harm himself and did not hear voices.

Following this hospitalization B.W. responded poorly to a behavioral incentive program and his antisocial behavior escalated. Within a couple of months he had several very serious altercations. He set fire to a piece of furniture in his home and to multiple items in the church his family attends, because he "doesn't like to go to church." He was apprehended by police during an attempted break-in-and-entry where he was alone and in possession of a hammer, a box cutter, and a lighter. He assaulted his principal and then resisted the arresting officer. He began stealing and lying constantly without remorse. He threatened his mother with a knife. Of most concern to the family was a malicious attack on his father. On the night before the attack he had to be restrained by his father for fear of hurting a sibling. To revenge the unwanted restraint he snuck up from behind his father the following day and delivered a blow to his father's head with a crescent wrench in a planned attack. His father said the most concerning aspect of the episode was that he did it "in cold blood, without any emotion."

### Neurology Evaluation

From age six to eleven B.W. had sporadic clusters of complex partial seizures occurring once every several months. The seizure frequency increased in parallel with the rise in behavioral problems in the months following psychiatric hospitalization at age eleven, prompting reevaluation by pediatric neurology (where he was seen again by A.D.B. along with C.J.). An MRI at this time showed evidence of a previously undetected vmPFC malformation (described in next section). He underwent an extensive diagnostic evaluation in an attempt to detect the seizure focus, including two inpatient admissions at major university hospitals. Video EEG monitoring captured 7 seizures with evidence of left frontal anterior-temporal onset in four, right-sided onset in one, and unclear laterality in two others. An ictal SPECT study showed increased perfusion diffusely in the left hemisphere, suggestive of a left-sided seizure focus but not localized further.

### Neuroimaging Results

**Figure 1:**
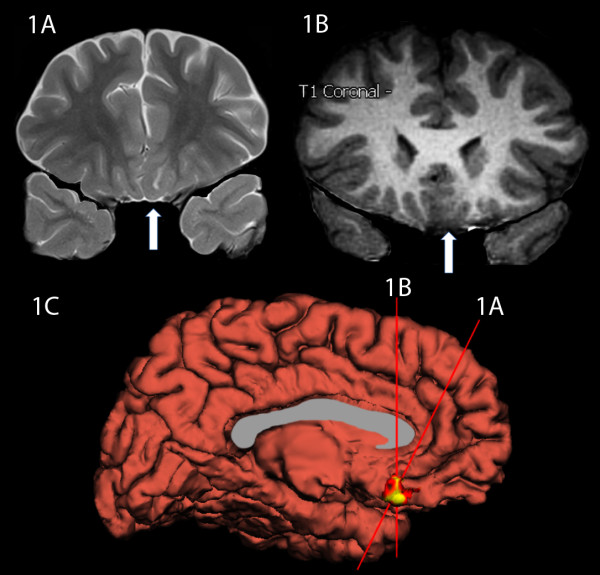
**MRI Images**. **1A**. This is an oblique coronal T2 image at the level immediately anterior to the horn of the lateral ventricles. Note the hyperintense white matter just deep to the gyrus rectus (indicated by arrow) with a linear extension tapering as it courses toward the anterior horn of the ventricle. Also note the cortical thickening of the left gyrus rectus relative to the right gyrus rectus. **1B**. This coronal T1 MPRAGE image shows thickening of the left ventromedial prefrontal cortex and blurring of the gray-white interface in this same region. **1C**. This is a surface rendering of B.W.'s brain viewing the medial left hemisphere surface with thickened cortex highlighted, which approximates the lesion site. See the appendix for further information on 1C.

Three of B.W.'s MRI studies of the brain were reviewed in detail and form the basis for our description. Two 1.5 Tesla (T) MRI examinations were done for clinical indications at ages four and eleven years, and a 3.0T MRI scan was done at age thirteen years for research purposes. The main finding was persistent and stable increased T2-signal intensity in the subcortical white matter of the left gyrus rectus in the vmPFC. This abnormal signal was best seen on thin section coronal T2-weighted and FLAIR sequences, and did not enhance following contrast administration (Figure 1A). Linear extension and tapering of the T2-hyperintense abnormality towards the frontal horn of the left lateral ventricle presumably reflects a radial neuronal migration line. Associated focal abnormal thickening of the cortex and blurring of the gray-white matter junction along the gyrus rectus were also evident (Figure 1B). These findings were stable between the patient's three available MRI studies. This constellation of MRI findings was thought to be compatible with Taylor type focal cortical dysplasia (FCD) with balloon cells, though confirmation of this subtype of FCD was not confirmed on histological analysis (see below) [33,34]. No other abnormalities of the brain were identified. The region of affected cortex is displayed in Figure 1C using thickness data generated from FreeSurfer software http://surfer.nmr.mgh.harvard.edu/. The lesion appears to involve a portion of Brodmann areas 11, 12, 25, and 32. See appendix for details regarding the generation of Figure 1C.

### Therapeutic Attempts\Surgical Resection

Therapeutic interventions for B.W.'s behavioral problems have included brief attempts at counseling beginning at age six and again at age nine, behavioral incentive therapy at age 11, and a panoply of medication trials. B.W. eventually underwent invasive grid mapping and subsequent resective epilepsy surgery in the summer of 2011. After extensive invasive mapping of the left ventromedial prefrontal region and also the left temporal area using grids and depth electrodes he underwent resection of the left prefrontal region as well as the mesial and lateral temporal structures. Pathology confirmed the presence of dysplastic neurons in clusters as well as scattered in the white matter of the left ventral prefrontal region and left amygdala, including surrounding mesial temporal cortex. The left amygdala region had been silent on MRI, however microscopic dysplastic neurons have been well described in other cases of malformations of cerebral cortex. B.W. remains seizure free on lamotrigine as the only drug for seizure control.

### Behavioral and Neuropsychological Testing

In addition to an extensive review of B.W.' s medical records, interviews with the family, and careful review of a detailed journal the parents kept detailing B.W.'s problem behavior we also obtained further behavioral and neuropsychological data. Informed consent was obtained in accord with the policies of the Institutional Review Board of the University of Iowa Carver College of Medicine. To serve as an intra-family comparison group we also obtained behavioral results for the siblings when possible (newborn and toddler excluded). All testing was performed when B.W. was 13, prior to surgical resection of the vmPFC lesion.  

#### Behavioral Questionnaires

B.W.'s parent(s) filled out the Child Behavior Checklist [35], the Antisocial Process Screening Device [36], and a version of the Iowa Scales of Personality Development (ISPD) [23] developed specifically for children with brain lesions [37]. The CBCL is a broad parent-completed questionnaire that examines a broad spectrum of childhood behavioral and emotional problems. The APSD is a parent-completed screening tool to evaluate for affective and interpersonal character traits of psychopathy. The ISPD is a tool developed to assess behavioral, emotional, and personality traits following childhood-onset brain damage. Items commonly associated with bilateral vmPFC damage are shown in bold. Specifically these items were rated as acquired disturbances (defined as a rating > 5) by over 70% of patients with bilateral vmPFC damage or were significantly higher in patients with vmPFC damage relative to non-prefrontal injuries (p < 0.001) [23]. The results of the CBCL, APSD, and ISPD are shown in Table 1. Superscripts in B.W.'s ISPD ratings denote statements and clarifications provided by B.W.'s mother.

**Table 1:** Behavioral Questionnaire Results

			**Siblings**		**B.W**.
	
	**8 yo M**	**11 yo F**	**15 yo F**	**Sibling Mean**	**13 yo**
	
**Child Behavior Checklist**					
Anxious/Depressed	50 (< 50th)	50 (< 50th)	70 (98th)**	57 (50-70)	60 (84th)
Withdrawn/Depressed	50 (< 50th)	50 (< 50th)	63 (89th)	54 (50-63)	66 (94th)*
Somatic Complaints	50 (< 50th)	51 (54th)	53 (58th)	51 (50-53)	58 (78th)
Social Problems	50 (< 50th)	51 (54th)	50 (< 50th)	50 (50-51)	69 (97th)*
Thought Problems	50 (< 50th)	50 (< 50th)	50 (< 50th)	50 (50-50)	71 (99th)**
Attention Problems	57 (75th)	51 (54th)	50 (< 50th)	53 (50-75)	65 (93rd)*
Rule-Breaking Behavior	50 (< 50th)	55 (69th)	50 (< 50th)	52 (50-55)	69 (97th)*
Aggressive Behavior	50 (< 50th)	60 (84th)	51 (54%)	54 (50-60)	72 (> 99th)**
Internalizing Composite	41	39	66	49 (39-66)	63
Externalizing Composite	40	59	47	49 (40-59)	71**
Total Problem Behavior	43	48	51	47 (43-59)	70*
**Antisocial Process Screening Device (APSD)**					
Callous-unemotional	1 (T = 42)	1 (T = 44)	1 (T = 44)	1 (T = 42-44)	9 (T = 79, 99.5%)**
Narcissism	3 (T = 49)	6 (T = 64)	6 (T = 47)	5 (T = 57-60)	12 (T = 85, 99.5%)**
Impulsivity	4 (T = 49)	3 (T = 47)	3 (T = 53)	3 (T = 43-47)	10 (T = 78, 100%)**
Total	11 (T = 51)	10 (T = 52)	10 (T = 50)	10 (T = 49-52)	32 (T = 87, 99.8%)**
**Iowa Scales of Personality (ISPD)**					
**Irritability**	3	6	3	4.0	7
**Lack of Persistence**	5	2	2	3.0	7 ^1^
**Lack of Insight**	2	3	1	2.0	7
**Poor Judgement**	2	2	1	1.7	7
**Unemotional**	2	2	5	3.0	7 (& 1) ^2^
Aggression	1	3	1	1.7	7
**Moodiness (lability)**	3	5	5	4.3	7^3^
**Inflexibility**	2	7	4	4.3	7
**Lack of Planning**	5	3	1	3.0	7^4^
**Inappropriate Emotion**	3	5	2	3.3	7^5^
Impulsivity	3	4	1	2.7	7
**Impatience\Poor**	5	5	3		7
**frustration tolerance**				4.3	
**Perseveration\Behavioral rigidity**	4	1	4		7
**Social**				3.0	
**Inappropriateness**	2	3	1		6^6^
**Indecisiveness**	3	2	5	3.3	6
**Lack of Initiative**	3	1	5	3.0	6
Indecisiveness	3	2	5	3.3	6
Depression	2	4	5	3.7	6
Manipulativeness	3	4	1	2.7	5
Obsessiveness	2	3	5	3.3	5^7^
Overly Active Behavior	2	5	1	2.7	5^8^
Easily Overwhelmed	6	2	5	4.3	5
Social Withdrawal	1	2	5	2.7	5^9^
Vanity	3	4	4	3.7	3
Suspiciousness	3	4	4	3.7	3
**Apathy**	3	1	5	3.0	3
Type A Behavior	1	6	3	3.3	3
Lack of Stamina	4	2	6	4.0	2^10^
Dependency	4	1	3	2.7	2^11^
Anxiety	3	1	7	3.7	1

*borderline clinical range, ** clinical range

ASPD T-Score Guideline: Mean T score = 50 with s.d. 10. < 45 = Below average, (not antisocial). 45-55 = Average, 56-60 = slightly atypical, 61-65 = mildly atypical, 66-70 = moderately atypical, > 70 markedly atypical, or highly antisocial. APSD percentiles based on empirical data gathered from a normative sample in parent ratings of male children (N = 411)

^1 ^Without his ADHD medications even simple chores are impossible.

^2 ^He has very strong emotions in disproportionate situations. Asking him to do chores may set off a temper tantrum. He can be giddy about small gifts or treats. He also may show very little emotion when he should express appropriate responses he may be flat, even if discussing brain surgery for himself.

^3 ^He is more likely to get into a funk that lasts hours over something that didn't go his way- then spiral in and out of rage.

^4 ^He lives only in the immediate.

^5 ^He'll burst into tears and have hours of sulking over not getting his way or if asked to do simple chores. However he does not display empathy or compassion towards others even if he has been the cause of distress.

^6 ^On ADHD medication he shows some restraint but on occasions when he has skipped them he's extremely inappropriate (eating with hands instead of utensils, noises, annoyances, etc)

^7 ^At times he can be haphazard and at others he'll spend hours on minutiae. For example, he gives very little attention to detail on school projects that do not hold his interest (i.e. reading, music) or require tenacity (sticking to commitments is a big problem for him). Conversely, he will spend hours tidying up his room or coloring a paper.

^8 ^Things that keep him occupied are limited (sports on TV, video games, puzzles, crafts/workshop). He does not read or enjoy board games. He prefers tangible activities.

^9 ^He wants to interact and asks for socializing but then seems incapable of participating.

^10 ^He is high energy but his attention span might prevent him from enduring things he finds tedious or boring

^11 ^He prefers to do things himself- almost to a fault in self-assessment of his capabilities (I'm going to build a tree house this afternoon).

Based on B.W.'s propensity for acquiring material items we also evaluated whether he had pathological collecting behavior using a standardized questionnaire, which has been used previously to demonstrate pathological collecting behavior in vmPFC patients [38]. We found that B.W. did collect quarters and pocket knives, but not extensively and the parents never considered this to be out of the ordinary. The parents reported that B.W. had difficulty throwing unneeded items away and had a tendency to accumulate useless items in Tupperware containers in his room (e.g. parts of watches, pieces of broken chairs, disassembled bike parts, buttons, ect.). The mother would periodically dispose the items so they never accumulated. He did not hoard food items. No collecting behavior data was gathered for the siblings.

#### Neuropsychological Testing

B.W. completed additional neuropsychological testing to assess his executive functioning abilities. On testing, he showed average set-shifting skills on the Trail-Making Test [39]. On the Wisconsin Card Sorting Task [40], he did not have difficulty with nonverbal concept formation or set-shifting. Perseverative errors and responses were minimal (superior range on both indices). Verbal fluency skills were solidly average on the Controlled Oral Word Association Test (COWA; [41]) and no set- losses or perseverative tendencies were observed. However, his performance on a design fluency task was somewhat perseverative. On a measure of attention, flexibility and ability to inhibit responses (Stroop Color and Word Test; [42], B.W. showed a normal performance across subtests. He had some difficulty with thinking ahead and planning on the Tower of Hanoi [43]. Specifically, once he got the correct concept on this task, he was not able to consistently use the information to repeat the task. Learning from consequences was also a weakness for B.W. His performance on the Iowa Gambling Task [44] suggested no learning and he was not able to determine which were good versus bad decks.

#### Moral Judgement

*B*.W. completed the Kohlberg Moral Judgement Task [45] and he and his siblings completed a paper-and-pencil version of the moral\convention distinction task [46]. Responses on the Kohlberg Moral Judgment Task suggested a relatively immature, preconventional, stage of moral development, in which moral dilemmas were approached primarily from the perspective of avoiding negative consequences for one's self. In the moral\convention distinction task B.W. and all of his siblings appropriately labeled moral transgressions as worse than conventional ones, particularly those causing physical pain to others. Qualitatively B.W. cited pain as a reason for not punching someone in the face, and for a story involving pulling a girls hair he stated that 'boys should never hurt a girl.' In terms of moral transgressions that damage property B.W. cited possible detention as the reason why it would not be acceptable to break a swing on the playground. It is noteworthy that all the siblings completed the task including those younger than B.W. On his first attempt, B.W. skipped several questions and scribbled over the entire second sheet and drew a goblin. He completed the task at a later date.

## Conclusions

To summarize, B.W. is a 14 year-old boy with a congenital malformation of the left vmPFC and microscopic evidence of dysplasia in the left amygdala and adjacent anteromedial temporal cortex. He had complex partial epilepsy that appears to be well- controlled following surgical resection. The most troublesome manifestation of the brain lesion for the family is the behavioral syndrome that has accompanied it. Central features of his history include: emotional lability, a lack of empathy, impulsivity, hyperactivity and an ability to manipulate others in order to indulge his own self-interests, which often center on acquiring material possessions. This includes persuading friends to steal for him and using a psychiatric hospitalization as leverage toward highly desired items. He gets angry and is prone to outbursts when people prevent him from acquiring those objects that he seeks. He displays a profound irreverence toward authority figures. While rare, he has also displayed the capacity for planned violence carried out in a cold, calculated manner.

The evidence supporting a causative relationship between the vmPFC lesion observed on MRI and the behavioral syndrome includes the following: 1) The behavioral and neuropsychological profile described in the results section is strikingly consistent with prior cases of focal vmPFC lesions. This is most apparent in reviewing the ISPD results. The severity of behavioral problems is more extreme than previously reported following vmPFC damage but this may represent an extension of prior reports of more severe outcomes following early-onset lesions [26]. It is interesting that B.W. like other vmPFC patients tends to perform relatively normally on standardized neuropsychological assessment. It may be because these standardized laboratory tests are not affected by profound egocentricity, but this seems to be a core feature of B.W.'s behavior that is obvious in observing real-world behavior. 2) There is a complete absence of externalizing and antisocial behavioral problems in B.W.'s family, suggesting a lower likelihood of a genetic predisposition. This is notable considering the high heritability of externalizing behavior, more generally [47,48], and the remarkably high heritability of callous and unemotional psychopathy, which B.W. shares several features [49]. 3) B.W. has exceptionally few social risk factors. He has intelligent, extraordinarily caring and motivated parents. They are raising his five siblings without behavioral problems. 4) One could argue that microscopic dysplastic tissue of the left amygdala and anteromedial temporal cortex may also have contributed significantly to B.W.'s behavioral problems. It is very common to identify pathological tissue in these anteromedial temporal structures in surgical resection for complex partial epilepsy but exceedingly uncommon to have such dramatic social impairments. For these reasons we believe it is safe to attribute the severe behavioral impairment to B.W.'s vmPFC malformation or the combination of the vmPFC and anteromedial pathology, but difficult to argue in favor of the anteromedial pathology being the primary basis of his behavioral problems.

### Delayed Behavioral Manifestations

Considering the congenital nature of the vmPFC lesion it is interesting that no sequelae were noted until age 4 with the onset of seizures. Like other patients with early-onset vmPFC lesions, B.W. has seemed to 'grow into' his behavioral impairments, with the first problems noted at 6 years of life and progressively worsening in later childhood and early adolescence. The late onset of behavioral problems may be secondary to the vmPFC being a late developing region that does not contribute significantly to behavior in early childhood. More likely there were subtle differences present earlier in life that were not detectable without the rigors of controlled experimental paradigms, as has been reported for lesions to the dorsolateral PFC [50]. Moreover, the behavioral deficits likely become more apparent as the social environment of late childhood and adolescence becomes more complex and expectations advance. It will also be interesting to see how the behaviors manifest differently as puberty progresses.

### Early injury and worse outcome

Brain injuries occurring early in life are often associated with better outcomes [51]. It seems the opposite is true for insult to the vmPFC [26,52,53]. These individuals tend to display more severe social misconduct and display impaired moral judgment. One hypothesis is that this is because distant brain areas depend on an intact interaction with the vmPFC to promote normal development. If this is true then a localized lesion to the vmPFC actually alters a large array of brain areas that are functionally connected to this region including the contralateral vmPFC, as reported in animal studies [54,55] and suggested by abnormal blood flow in the prefrontal lobe bilaterally following unilateral left prefrontal injury at seven years of age [21]. A functional corollary to this possibility is that the vmPFC may have a role in internally modeling the emotional dynamics of one's environment. Upon forming associations between the contexts of different situations and the emotional tone of that situation the vmPFC may work with other brain areas to consolidate these associations. If the vmPFC is damaged later in life the person may be better equipped to compensate via experiences gained with an intact vmPFC. With this in mind it is interesting to compare B.W.'s behavioral problems to adult-onset lesions of similar size and location. Two cases described by Tranel et al in 2005 (patient 1652 and 0297) are close approximations of the extent of vmPFC lesion, including the location and laterality [56]. The real-world behavioral deficits in these patients is strikingly mild to absent and contrast starkly with B.W.'s profile. A limitation to this comparison is that B.W. has microscopic anteromedial temporal lobe pathology, which is presumed absent from these two adult patients. The deficits seen in association with early-onset lesions likely provides a more accurate view of what the vmPFC contributes to the development of human social behavior.

### Lesion laterality

Historically it was postulated that a bilateral lesion to the vmPFC was required to severely disrupt social behavior [12]. The minimal requisite damage was challenged by case reports showing unilateral injury associated with personality changes and antisocial behavior [20,21]. It has also been noted that when vmPFC damage is unilateral it tends to be the right-sided lesions that are more detrimental in males [56]. In the current case there is only radiographic evidence of a left-sided lesion. This would appear to challenge the notion of right vmPFC being preferentially important for social behavior in males, considering the severity of BW's impairments. As alluded to above it is possible the contralateral vmPFC (right-side) developed abnormally secondary to abnormal input stemming from the left-sided lesion. It is also possible that the combination of the left vmPFC and left anteromedial temporal lobe pathology is critical in this case.

### Is B.W. a pyschopath?

Similarities of psychopathy and 'acquired psychopathy' following vmPFC injury have been reported in several domains: behavioral, neuropsychological and psychophysiologic, as reviewed in [57]. As a result of this work the vmPFC figures prominently in theoretical frameworks for understanding the neurobiology of psychopathy [6,58]. Differences have also been noted, such as a lack of overt violence or criminal behavior in acquired psychopathy [11,59,60]. Proponents of the vmPFC as a causative factor in psychopathy hypothesize that the differences in developmental psychopathy and vmPFC injury stem from the time of vmPFC dysfunction, either congenital (in the case of developmental psychopathy) or acquired later in life at the lesion onset. Studies of early-onset vmPFC damage support this notion, with a profile closer to that seen in developmental psychopathy relative to their late-onset vmPFC lesion counterparts [26]. In this study we are in a unique position to address this topic because the lesion is congenital. We used the antisocial process screening device, a tool to evaluate "psychopathic tendencies" in children. B.W. scored high in all domains of the test with an overall score in the 99.8 percentile. The authors of this screening tool caution against applying the label psychopath to any child because of its derogatory connotations. In this regard we agree and will instead say that B.W. shares several of the interpersonal and affective characteristics commonly seen in developmental psychopathy.

To conclude, this case adds to a rich literature on the consequences of vmPFC damage and extends it by further highlighting similarities to developmental psychopathy seen when unilateral vmPFC dysfunction occurs as a result of a congenital lesion. Despite increasingly sophisticated observations and behavioral testing paradigms used to describe the deficits in individuals with vmPFC lesions we still lack a satisfactory explanation for exactly what this sector of brain tissue contributes to development. Further investigation is warranted. Improved understanding of the underlying mechanisms by which vmPFC damage leads to behavioral dysfunction described above may ultimately advance the therapeutic modalities available for vmPFC patients as well as in psychiatric conditions with vmPFC involvement.

## Abbreviations

APSD: Antisocial process screening device; CBCL: Child Behavior Checklist; ISPD: Iowa scales of personality development; vmPFC: Ventromedial prefrontal cortex.

## Competing interests

The authors declare that they have no competing interests.

## Authors' contributions

ADB was the primary author of the manuscript. He reviewed medical, personal, and neuropsychological reports of BW as well as interviewed the parents. AHG was involved in the neuropsychological and behavioral testing of BW and BW's siblings. This included analyzing the results of these studies. CJ is the primary neurologist of BW and reviewed his neurological course. NC reviewed the MRI data and contributed to the description of the neuroimaging findings. PN was involved in the production of the FreeSurfer image displaying BW's lesion as well as reviewing the manuscript. SWA was a senior author on the paper and oversaw all aspects of the paper, including a careful review of the final product. All authors read and approved the final manuscript.

## Appendix

The FreeSurfer software package was used to provide a visual demonstration of the affected vmPFC cortical region on a surface representation of B.W.'s brain (see Figure 1C). FreeSurfer has been validated and described in detail elsewhere http://surfer.nmr.mgh.harvard.edu/[61-63] and was used to delineate the cortical mantle. In brief, the cortical mantle was determined by segmenting white matter, tessellating the surface representation along the gray-white matter junction, and inflating until the pial surface is approximated. The gray-white matter and the pial surface representations were then refined based on intensity and continuity information using a deformable surface algorithm. For each vertex on the tessellated surfaces, cortical thickness was calculated based on the average of the shortest distance from pial surface to the gray/white surface and vice versa [64]. Absolute cortical thickness was displayed on a surface representation of B.W.'s brain with a color scale demonstrating vertices in warm colors if they are in the upper 5th percentile of thickness values. This image included the region of the vmPFC shown in Figure 1C. Anteromesial temporal lobe and temporal pole, which are the thickest regions of the human cerebral cortex, were also displayed in this initial thickness map. The thickness display from this map in the region of vmPFC was overlaid onto an otherwise plain surface representation of B.W.'s brain using Adobe Photoshop to highlight the affected region of cortex to the exclusion of the anteromesial temporal region which is presumed to have normal cortical thickness.

## Pre-publication history

The pre-publication history for this paper can be accessed here:

http://www.biomedcentral.com/1471-2377/11/151/prepub
